# East Meets West: Community-Based Programs for Dementia Prevention and Support

**DOI:** 10.1093/geroni/igab046.1871

**Published:** 2021-12-17

**Authors:** Fei Sun, Melissa Batchelor

## Abstract

This East Meets West Symposium presents cross-cultural evidence of community-based programs developed to address cognitive health needs of older adults or to support dementia family caregivers. It includes five studies from mainland China, Taiwan, and the U.S.A. The first study explored the adherence to computerized cognitive training to promote cognitive health among Chinese older adults using a qualitative approach. External encouragement, and self-awareness of performance improvement were found influential to older adults’ adherence. The second study spearheaded by American Association of Retired Persons (AARP) in the U.S.A shed further light on technology use in promoting brain health by comparing in-person versus online cognitive training to older Americans during the COVID-19 pandemic. The third study tested the effectiveness of a cognitive health management model for community-dwelling older adults including healthy and mild cognitively impaired (MCI) ones in Shanghai, China. Older adults with MCI who received the management service showed better cognition outcomes compared to their counterparts in the control group. The fourth study focused on a community program named “Carer Café”, developed to support dementia family caregiver in Taiwan. Participants (n=375) reported reduced stress and increased access to service referrals. The last study examined the stakeholders’ perceptions of dementia friendly initiatives (DFI) being implemented in U.S.A, mainland China, and Taiwan to support persons with dementia and family caregivers. The impacts of DFI made, challenges DFI faced, and strategies DFI used during COVID-19 pandemic were discussed. The discussant will comment on each article’s contributions and limitations in the context of literature.

